# Supplementary material for: Serotonergic neurons regulate the Drosophila vascular niche to control immune stress hematopoiesis
Source: Nat Commun. 2025 Jun 3;16:5152. doi: 10.1038/s41467-025-60493-y (PMC12134330; doi:10.1038/s41467-025-60493-y)
Supplement: Supplementary file 1 — Supplementary Information [file 41467_2025_60493_MOESM1_ESM.pdf]

Sup. Fig. 1

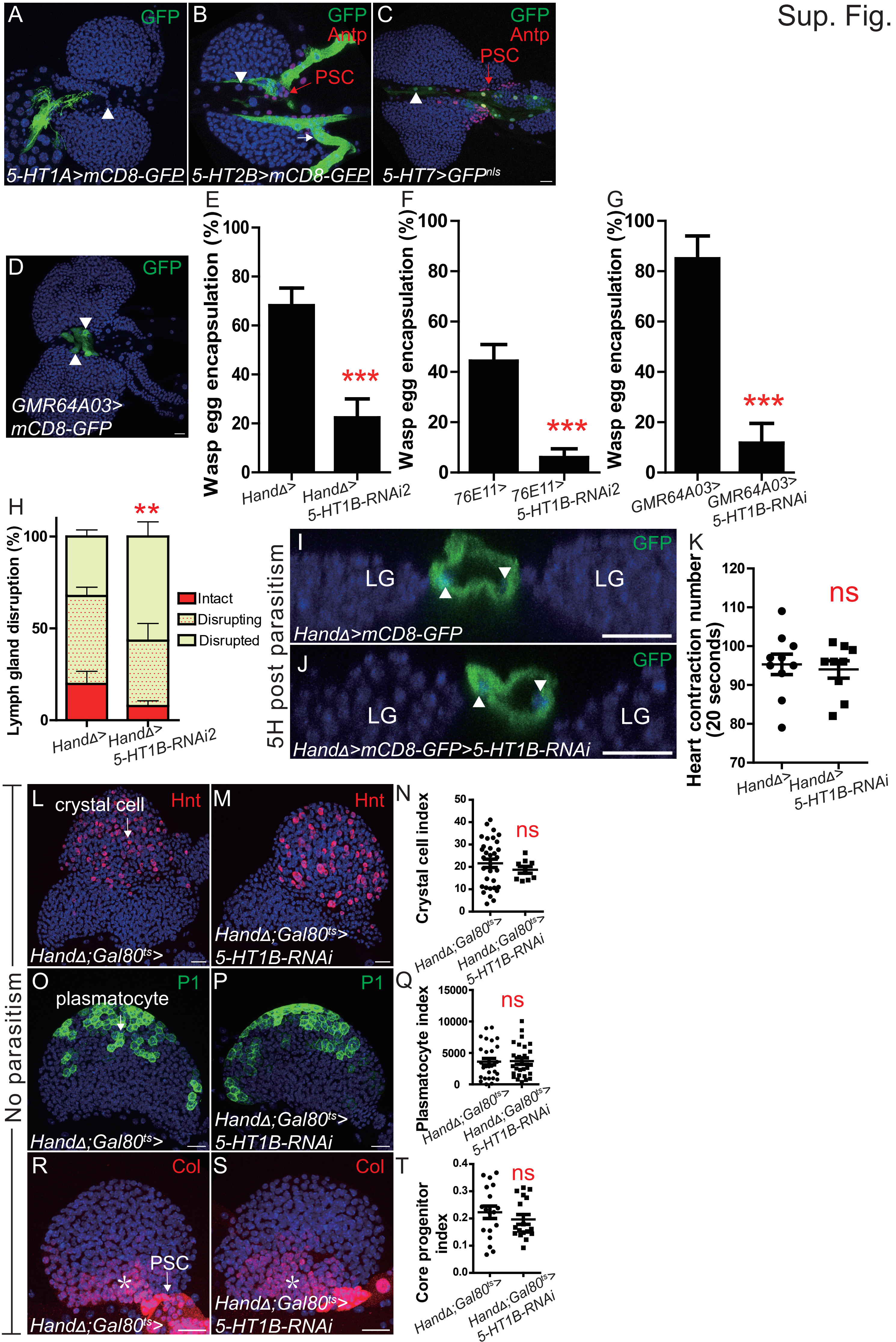

A
GFP
5-HT1A>mCD8-GFP
B
GFP
Antp
PSC
5-HT2B>mCD8-GFP
C
GFP
Antp
PSC
5-HT7>GFP^nls
D
GFP
GMR64A03>
mCD8-GFP
E
Wasp egg encapsulation (%)
100
80
60
40
20
0
***
HandΔ>
HandΔ>
5-HT1B-RNAi2
F
Wasp egg encapsulation (%)
***
76E11>
76E11>
5-HT1B-RNAi2
G
Wasp egg encapsulation (%)
***
GMR64A03>
GMR64A03>
5-HT1B-RNAi
H
Lymph gland disruption (%)
**
Intact
Disrupting
Disrupted
HandΔ>
HandΔ>
5-HT1B-RNAi2
5H post parasitism
I
GFP
LG
LG
HandΔ>mCD8-GFP
J
GFP
LG
LG
HandΔ>mCD8-GFP>5-HT1B-RNAi
K
Heart contraction number (20 seconds)
ns
120
110
100
90
80
70
HandΔ>
HandΔ>
5-HT1B-RNAi
No parasitism
L
crystal cell
Hnt
HandΔ;Gal80ts>
M
Hnt
HandΔ;Gal80ts>
5-HT1B-RNAi
N
Crystal cell index
ns
HandΔ;Gal80ts>
HandΔ;Gal80ts>
5-HT1B-RNAi
O
plasmatocyte
P1
HandΔ;Gal80ts>
P
P1
HandΔ;Gal80ts>
5-HT1B-RNAi
Q
Plasmatocyte index
ns
15000
10000
5000
0
R
Col
PSC
HandΔ;Gal80ts>
S
Col
HandΔ;Gal80ts>
5-HT1B-RNAi
T
Core progenitor index
ns
0.4
0.3
0.2
0.1
0.0

Sup. Figure 1: 5-HT1A, 5-HT2B and 5-HT7 serotonin receptor expression. 5-HT1B expression on anterior aorta cells is required for immune defense against wasp parasitism but is dispensable for steady state lymph gland hematopoiesis, cardiac tube morphology and beating

(A) *5-HT1A-Gal4(KI)>UAS-mCD8-GFP* is expressed in neurons innervating the ring gland. (B) *5-HT2B-Gal4(KI)>UAS-mCD8-GFP* is expressed in alary muscles (white arrow) and Antp (red) labels the PSC (red arrow). (C) *5-HT7-Gal4(KI)>UAS-GFP*^{nls} is barely detected in cardiac cells and Antp (red) labels the PSC (red arrow). There are no tools for analyzing the expression profile of the 5-HT2A receptor. Cardiac cell nuclei are indicated by white arrowheads. (D) *GMR64A03-Gal4* (*Creb-Gal4)>mCD8-GFP* is expressed in anterior aorta cells (green, white arrowhead) in between lymph gland anterior lobes. (E-G) Quantification (%) of wasp egg encapsulation using cardiac cell drivers *HandΔ-Gal4* (E), *76E11-Gal4* (F), and *GMR64A03-Gal4* (G). The mean of three independent experiments is represented: control n=113 (E), 132 (F), 62 (G) ; *5-HT1B-RNAi2* n=99 (E) and 107 (F), and *5-HT1B-RNAi* n=69 (G). (H) Quantification (%) of lymph gland disruption 13H post parasitism. Quantification represents the mean of three independent biological replicates: control n=66 and *5-HT1B-RNAi2* n=67. (I-J) Transversal sections of anterior aorta expressing a membrane-bound GFP (*HandΔ>mCD8-GFP*, green) in control (I) and *5-HT1B-KD* (*HandΔ>mCD8GFP>5-HT1B-RNAi)* (J). No difference in the lumen is observed. Lymph gland anterior lobe nuclei are visualized with DAPI. (K) Number of larval heart contractions per 20s. No difference is observed between *5-HT1B-KD (n=9)* and control (n=10). (L-M) Crystal cell differentiation (Hnt, red) in control (L) and in *5-HT1B-KD* (M). (N) Crystal cell index. (O-P) Plasmatocyte differentiation (P1, green) in control (O) and in *5-HT1B-KD* (P). (Q) Plasmatocyte index. (R-S) Col (red) labels core progenitors (*) and the PSC (white arrow) in control (R) and in *5-HT1B-KD* (S). (T) Core progenitor index. (L-T) At least three biological replicates were performed and one is shown. Error bars represent SEM. Statistical test: Mann-Whitney nonparametric test, two-tailed in K, N, Q, T and Pearson's Chi-squared test, one-tailed in E-H. ** $p<0.01$, *** $p<0.001$, ns non-significant. Scale bars, 20μm.

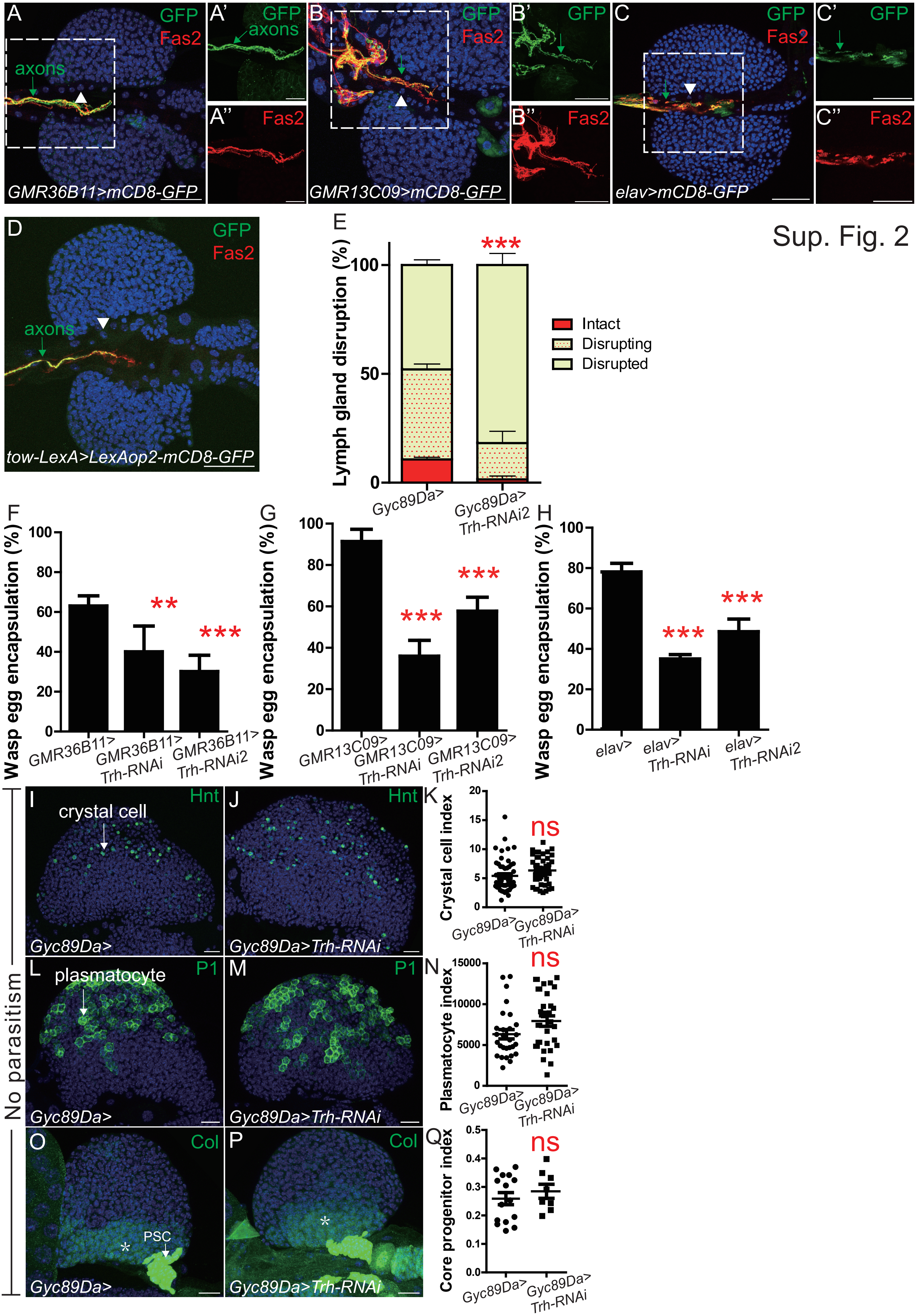

Sup. Fig. 2
A
GFP
Fas2
axons
GMR36B11>mCD8-GFP
A'
GFP
axons
A''
Fas2
B
GFP
Fas2
GMR13C09>mCD8-GFP
B'
GFP
B''
Fas2
C
GFP
Fas2
elav>mCD8-GFP
C'
GFP
C''
Fas2
D
GFP
Fas2
axons
tow-LexA>LexAop2-mCD8-GFP
E
Lymph gland disruption (%)
Intact
Disrupting
Disrupted
Gyc89Da>
Gyc89Da> Trh-RNAi2
***
F
Wasp egg encapsulation (%)
GMR36B11>
GMR36B11> Trh-RNAi
GMR36B11> Trh-RNAi2
**
***
G
Wasp egg encapsulation (%)
GMR13C09>
GMR13C09> Trh-RNAi
GMR13C09> Trh-RNAi2
***
***
H
Wasp egg encapsulation (%)
elav>
elav> Trh-RNAi
elav> Trh-RNAi2
***
***
No parasitism
I
Hnt
crystal cell
Gyc89Da>
J
Hnt
Gyc89Da>Trh-RNAi
K
Crystal cell index
Gyc89Da>
Gyc89Da> Trh-RNAi
ns
L
P1
plasmatocyte
Gyc89Da>
M
P1
Gyc89Da>Trh-RNAi
N
Plasmatocyte index
Gyc89Da>
Gyc89Da> Trh-RNAi
ns
O
Col
PSC
Gyc89Da>
P
Col
Gyc89Da>Trh-RNAi
Q
Core progenitor index
Gyc89Da>
Gyc89Da> Trh-RNAi
ns

Sup. Figure 2: Identification of Neurons^{aorta}-Gal4 and LexA drivers; Trh is required in neurons to regulate lymph gland rupture and wasp egg encapsulation following parasitism

(A-A”) *GMR36B11-Gal4>UAS-mCD8GFP* (green) and Fas2 (red) labels Neurons^{aorta} axons. (B-B”) *GMR13C09-Gal4>UAS-mCD8-GFP* (green; B-B’) and Fas2 (red; B, B”) labels Neurons^{aorta} axons. (C-C”) *elav-Gal4>UAS-mCD8-GFP* (green; C-C’) and Fas2 (red; C, C”) labels Neurons^{aorta} axons. (D) *tow-LexA>LexAop2-mCD8-GFP, GFP (*green) expressed in Neurons^{aorta} axons. White arrowheads indicate aorta nuclei. (E) Quantification of lymph gland disruption (%) 13H post parasitism. Quantification represents the mean of three independent biological replicates: control n=65, *Trh-RNAi2* n=66. (F-H) Quantification (%) of wasp egg encapsulation using as neuron driver *GMR36B11-Gal4* (F), *GMR13C09-Gal4* (G) or *elav-Gal4* (H) to express two distinct *Trh-RNAi.* The mean of three independent experiments is represented: control n=109 (F), 98 (G), 92 (H); *Trh-RNAi* n=87 (F), 102 (G), 57 (H) and *Trh-RNAi2* n=145 (F), 130 (G), 106 (H). (I-J) Crystal cell differentiation (Hnt, green) in control (I) and when *Trh* RNAi is expressed in neurons using *Gyc89Da-Gal4* driver *(Trh-KD)* (J). (K) Crystal cell index. (L-M) Plasmatocyte differentiation (P1, green) in control (L) and *Gyc89Da>Trh-RNAi* (M). (N) Plasmatocyte index. (O-P) Col (green) labels core progenitors (*) and the PSC (white arrow) in control (O) and *Gyc89Da>Trh-RNAi* (P). (Q) Core progenitor index. (I-Q) At least three biological replicates were performed and one is shown. Error bars represent SEM. Statistical test: Mann-Whitney nonparametric test, two-tailed in K, N, Q and Pearson’s Chi-squared test, one-tailed in E-H. ** $p<0.01$, *** $p<0.001$, ns non-significant. Scale bars, 20μm.

Sup. Fig. 3

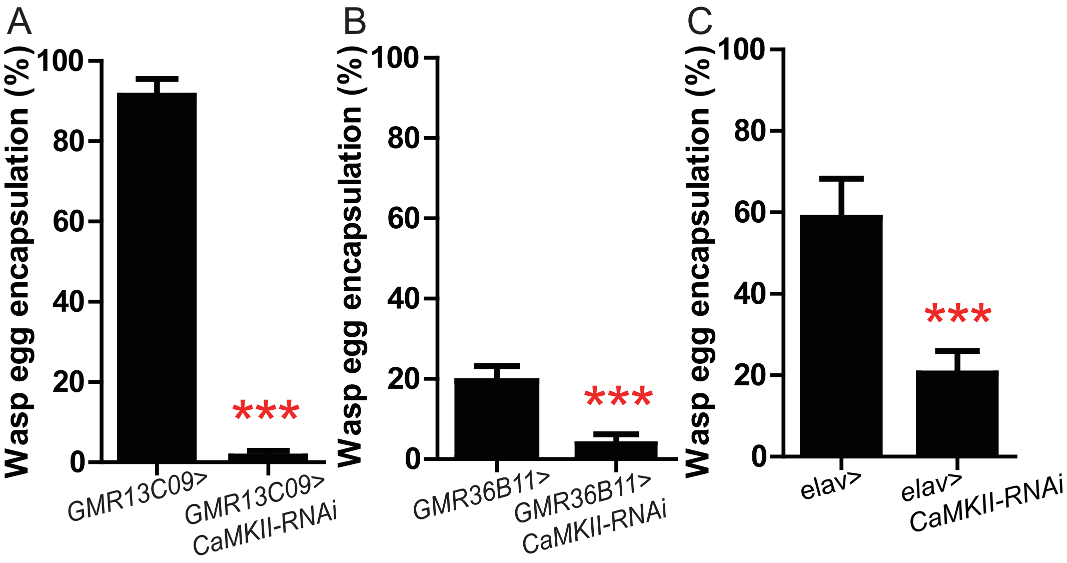
A
B
C
Wasp egg encapsulation (%)
100
80
60
40
20
0
***
GMR13C09>
GMR13C09>
CaMKII-RNAi
GMR36B11>
GMR36B11>
CaMKII-RNAi
elav>
elav>
CaMKII-RNAi

Sup. Figure 3: Neuronal activity is required for wasp egg encapsulation

(A-C) Quantification (%) of wasp egg encapsulation using as neuron driver *GMR13C09-Gal4* (A), *GMR36B11-Gal4* (B) or *elav-Gal4* (C) to express *CaMKII-RNAi.* The mean of three independent experiments is represented: control n=96 (A), 160 (B), 242 (C); *CaMKII-RNAi* n=82 (A), 122 (B) and 251 (C). Error bars represent SEM. Statistical test: Pearson's Chi-squared test, one-tailed. *** p<0.001.

Sup. Fig. 4

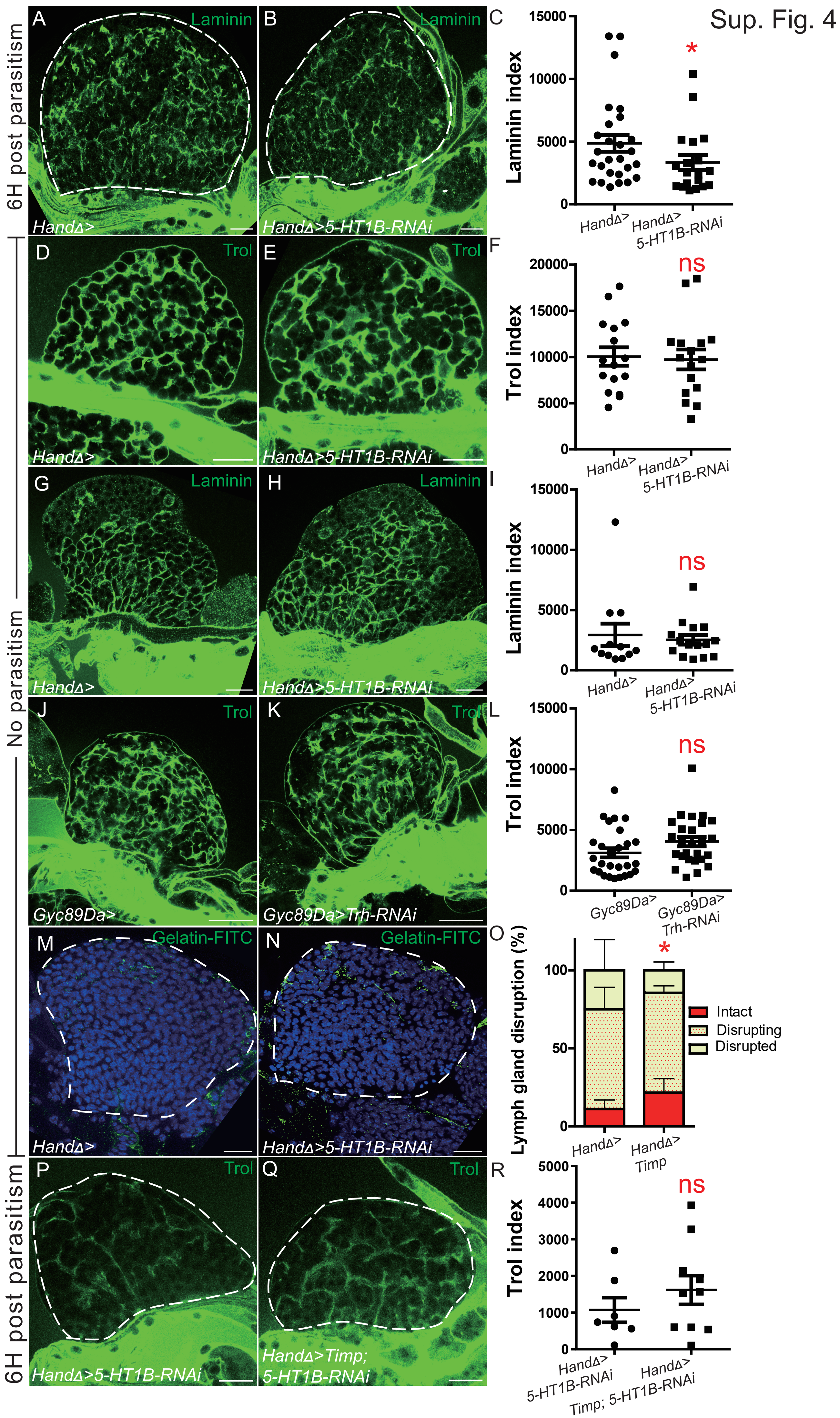

6H post parasitism
No parasitism
6H post parasitism
A Laminin
HandΔ>
B Laminin
HandΔ>5-HT1B-RNAi
C
Laminin index
15000
10000
5000
0
*
HandΔ>
HandΔ> 5-HT1B-RNAi
D Trol
HandΔ>
E Trol
HandΔ>5-HT1B-RNAi
F
Trol index
20000
15000
10000
5000
0
ns
HandΔ>
HandΔ> 5-HT1B-RNAi
G Laminin
HandΔ>
H Laminin
HandΔ>5-HT1B-RNAi
I
Laminin index
15000
10000
5000
0
ns
HandΔ>
HandΔ> 5-HT1B-RNAi
J Trol
Gyc89Da>
K Trol
Gyc89Da>Trh-RNAi
L
Trol index
15000
10000
5000
0
ns
Gyc89Da>
Gyc89Da> Trh-RNAi
M Gelatin-FITC
HandΔ>
N Gelatin-FITC
HandΔ>5-HT1B-RNAi
O
Lymph gland disruption (%)
100
50
0
*
Intact
Disrupting
Disrupted
HandΔ>
HandΔ> Timp
P Trol
HandΔ>5-HT1B-RNAi
Q Trol
HandΔ>Timp; 5-HT1B-RNAi
R
Trol index
5000
4000
3000
2000
1000
0
ns
HandΔ> 5-HT1B-RNAi
HandΔ> Timp; 5-HT1B-RNAi

Sup. Figure 4: Defect in Laminin network in 6H post parasitism; Trol and Laminin meshworks are not affected in *HandΔ>5-HT1B-RNAi* and *Gyc89Da>Trh-RNAi* under homeostatic conditions

(A-B) Laminin immunostaining (green) in control (A) and *HandΔ>5-HT1B-RNAi* (B) 6H post parasitism. (C) Laminin index in lymph gland anterior lobes. (D-E) Trol expression (green) in control (D) and in *HandΔ>5-HT1B-RNAi* (E) in the absence of parasitism. (F) Trol index in lymph gland anterior lobes. (G-H) Laminin expression (green) in control (G) and in *HandΔ>5-HT1B-RNAi* (H) in the absence of parasitism. (I) Laminin index in lymph gland anterior lobes**.** (J-K) Trol expression (green) in control (J) and in *Gyc89Da>Trh-RNAi* (K) in the absence of parasitism. (L) Trol index in lymph gland anterior lobes. (M-N) Zymography assay, gelatin-FITC (green) in control (M) and in *HandΔ>5-HT1B-RNAi* (N) in the absence of parasitism. (O) Quantification (%) of lymph gland disruption 13H post-parasitism for indicated genotypes. Quantification represents the mean of three independent biological replicates: control n=79, *Timp* n=80. (P-Q) Trol immunostaining (green) 6H post parasitism in *HandΔ>5-HT1B-RNAi* (P) and *HandΔ> Timp; 5-HT1B-RNAi* (Q) lymph glands. (R) Trol index in lymph gland anterior lobes. (A-L, P-R) Three biological replicates were performed and one is shown. L Error bars represent SEM. Statistical test: Mann-Whitney nonparametric test, two-tailed in C, F, I, L, R, and Pearson's Chi-squared test, one-tailed in O. * $p<0.05$, ns non-significant. Scale bars, 20μm.

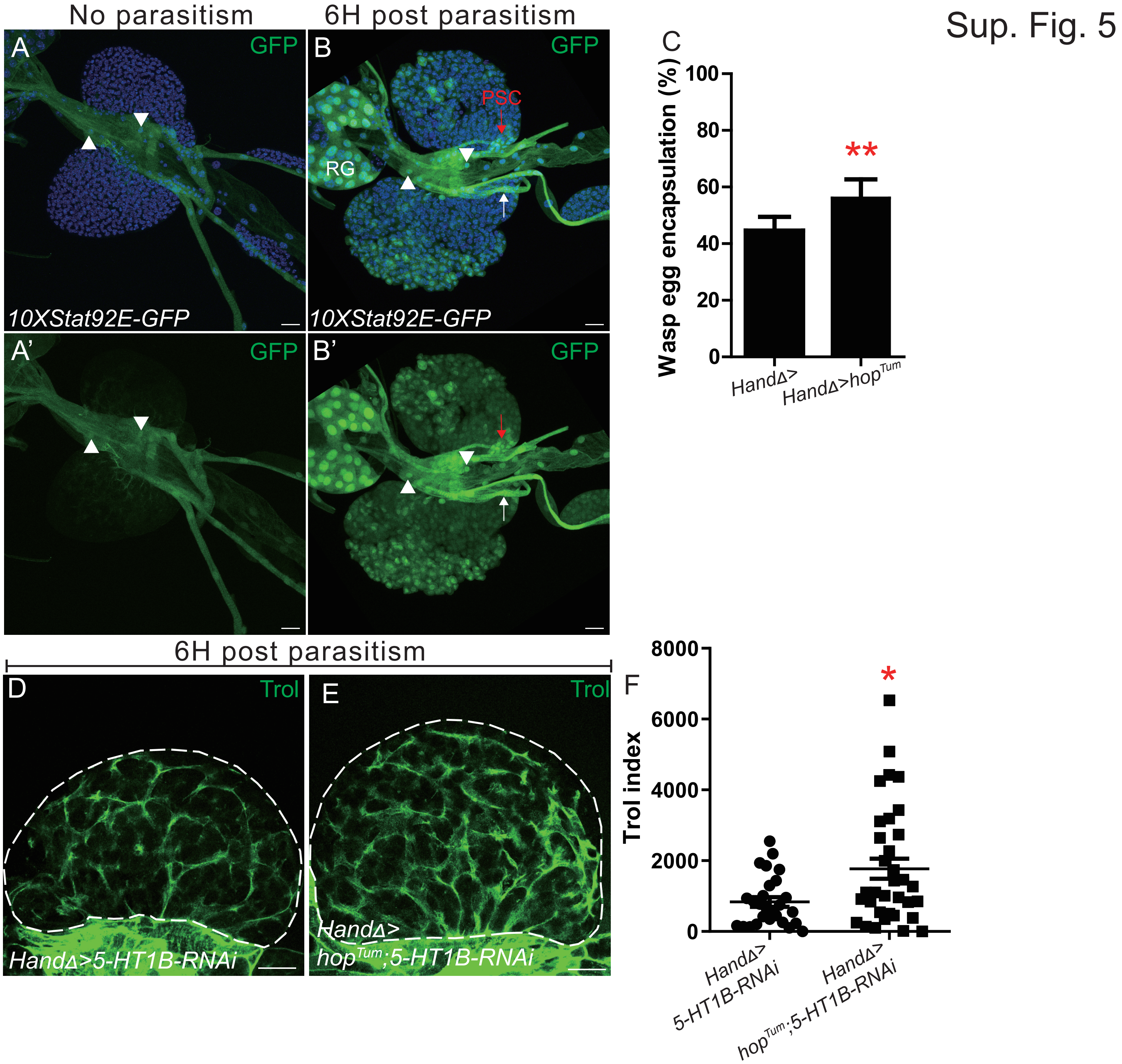

Sup. Fig. 5
No parasitism
6H post parasitism
A
B
GFP
PSC
RG
10XStat92E-GFP
A'
B'
C
Wasp egg encapsulation (%)
100
80
60
40
20
0
**
HandΔ>
HandΔ>hopTum
6H post parasitism
D
E
Trol
HandΔ>5-HT1B-RNAi
HandΔ>
hopTum;5-HT1B-RNAi
F
Trol index
8000
6000
4000
2000
0
*
HandΔ>
5-HT1B-RNAi
HandΔ>
hopTum;5-HT1B-RNAi

Sup. Figure 5: JAK/STAT signaling is activated in cardiac cells 6H post parasitism and is required to regulate wasp egg encapsulation

(A-B') Lymph glands from larvae expressing *10xStat92E-GFP* (green) without parasitism (A-A') and 6H post parasitism (B-B'). Post parasitism, *10xStat92E-GFP* expression is increased in cardiac cells (white arrowhead), in the PSC (red arrow), in the cortex of the lymph gland, in the alary muscle (white arrow), and in the ring gland (RG). (C) Quantification (%) of wasp egg encapsulation. The mean of four independent experiments is represented: control n=583; *hop*^{Tum} n=420. (D-E) Trol immunostaining (green) 6H post parasitism in *HandΔ>5-HT1B-RNAi* (D) and *HandΔ>hop*^{Tum}*; 5-HT1B-RNAi* (E) lymph glands. (F) Trol index in lymph gland anterior. Quantification corresponds to three independent biological replicates. Error bars represent SEM. Statistical test: Mann-Whitney nonparametric test, two-tailed in F and Pearson's Chi-squared test, one-tailed in C. * $p<0.05$, ** $p<0.01$. Scale bars, 20μm.
